# Effects of SP600125 and hypothermic machine perfusion on livers donated after cardiac death in a pig allograft transplantation model

**DOI:** 10.1186/s40001-020-00472-9

**Published:** 2021-02-05

**Authors:** Yijie Zhang, Qi Pan, Ying Cheng, Yongfeng Liu

**Affiliations:** 1 Department of Organ Transplantation and Hepatobiliary, The First Affiliated Hospital of China Medical University, No. 155, Nanjing Street, Shenyang, 110001 Liaoning People’s Republic of China; 2 The Key Laboratory of Organ Transplantation of Liaoning Province, The First Affiliated Hospital of China Medical University, No. 155, Nanjing Street, Shenyang, 110001 Liaoning People’s Republic of China

## Abstract

**Background:**

Hypothermic machine perfusion (HMP) improves the quality of donor livers for transplantation, both in animal models and in clinical practice. Treatment with SP600125, an inhibitor of c-Jun N-terminal kinase (JNK), can suppress the JNK signaling pathway to alleviate donor liver ischemia–reperfusion injury (IRI). We performed the present study with the objective of exploring the protective effects exerted by a combination of HMP and SP600125 on liver xenograft viability for donation after cardiac death (DCD) in a porcine model.

**Methods:**

54 adult BAMA mini-pigs were randomly assigned to 5 groups, including sham, cold storage for 4 h (CS 4 h), CS 4 h + SP600125, CS 2 h + HMP 2 h, and CS 2 h + HMP 2 h + SP600125 groups. Donor livers in the CS 4 h and CS 4 h + SP600125 groups were conventionally cold preserved for 4 h, whereas donor livers in the CS 2 h + HMP 2 h and CS 2 h + HMP 2 h + SP600125 groups were cold preserved for 2 h and then treated with HMP for 2 h. The preservation and perfusion solutions contained SP600125 (20 µM). Follow-up was conducted for 5 days after liver transplantation to compare the surgical outcomes by means of serological examination, pathological results, and survival rate.

**Results:**

The most satisfactory outcome after liver transplantation was observed in the CS 2 h + HMP 2 h + SP600125 group, which presented with minimal damage of donor livers during 5 days’ follow-up. Additionally, serological examination, pathological results, and survival rate concurred in showing better results in the CS 2 h + HMP 2 h ± SP600125 group than in the CS 4 h ± SP600125 group.

**Conclusion:**

HMP in combination with SP600125 has hepatoprotective properties and improves the quality and viability of porcine livers collected after DCD, thus improving prognosis after liver transplantation.

## Introduction

Liver transplantation is currently the only effective approach in the management of end-stage liver diseases. However, candidates for life saving liver transplant suffer an increased risk of mortality while waiting for an organ, due to the worldwide shortage of live liver donors [1, 2]. Therefore, liver donation after cardiac death (DCD) is increasingly applied in clinical practice [3, 4]. The viability of DCD livers depends critically on the duration and conditions of handling after donation. Hypothermic machine perfusion (HMP), which is currently the most advanced mode of organ perfusion, has been widely applied in kidney transplantation [5], and is likewise beneficial in donor liver preservation, owing to its ability to alleviate ischemia–reperfusion injury (IRI) and thus improve donor liver quality [6–9]. Recent studies in the field or organ transplant are focusing on the intracellular and extracellular signal transduction mechanisms related to IRI [10]. One of the key findings to date concerns the close relationship between mitogen-activated protein kinase (MAPK) and the incidence and development of IRI [11]. One of the downstream mediators of the MAPK signaling pathway is the c-Jun N-terminal kinase (JNK) pathway, which is activated by several stressors, including ultraviolet rays, heat shock, hypertonicity, and IRI, thus bringing the designation of the stress-activated protein kinase signaling pathway [12, 13]. At present, the significant participation of the JNK signaling pathway in liver IRI has been validated by numerous studies, which suggests that the suppression of the JNK signaling pathway might alleviate IRI of donor livers following liver transplantation, thus improving the prognosis in rat and mouse models [14–17], and by extension in the clinic.

SP600125 (1,9-pyrazoleanthrone, molecular formula: C_14_H_8_N_2_O, molecular weight: 220.23) effectively exerts selective inhibition on JNK1, JNK2, and JNK3. The cascade reaction of the JNK signaling pathway can be suppressed by SP600125, which competes against ATP binding to inhibit the phosphorylation of c-Jun, leading to its inactivation. The application of SP600125 to inhibit the JNK signaling pathway can alleviate IRI of donor livers following liver transplantation in rodent models [14, 15, 17, 18]. However, the alleviation of IRI through of SP600125-dependent inhibition of the JNK signaling pathway requires further verification and research with larger-bodied animals, among which the pig is widely used in transplantation research. While the toxic side effects of SP600125 have limited its use for human organ donors or recipients, we suppose that its application as an adjunct in the HMP perfusion medium might circumvent its systemic toxicity, while imparting better survival of the transplanted organ.

To test this hypothesis, we undertook for the first time a study in pigs for the verification of the protective effects of HMP combined with SP600125 in the perfusion solution on donor livers. Since aspects of porcine biology and physiology are similar to humans [19], findings of a protective action of HMP and SP600125 in liver donation after cardiac death may be directly translatable for clinical practice in human liver failure patients.

## Materials and methods

### Experimental animals

54 healthy adult BAMA pigs (age: 10–12 months, weight: 30–40 kg; Shenyang Agricultural University, Shenyang, Liaoning, China) of either gender were enrolled for model establishment. The animal experiments were approved by the Animal Care and Use Committee of the First Affiliated Hospital of China Medical University (No. 20190512) and were performed in accordance with the guidelines of the Provincial Laboratory Animal Science Association of Liaoning Province.

### Experimental design

The pigs were numbered 1–54, weighed, and renumbered based on their body weight. The pigs were divided into 5 groups: in (i) the sham group (*n* = 6), the were anesthetized, the abdomen was incised and perihepatic ligament mobilized, followed by extubation for 2 h after abdominal closure, (ii) the WIT 30 min + CS 4 h DCD group (*n* = 12), in which the pigs were subjected to warm ischemia for 30 min and the excised livers cold preserved for 4 h, followed by liver transplantation, (iii) the WIT 30 min + CS 4 h + SP600125 DCD group (*n* = 12), which were subjected to warm ischemia for 30 min and cold preserved for 4 h, with addition of 20 µM SP600125 into the preservation solution, followed by liver transplantation), (iv) the WIT 30 min + CS 2 h + HMP 2 h DCD group (*n* = 12), which were subjected to warm ischemia for 30 min and cold preserved for 2 h, followed by HMP for 2 h and liver transplantation, and (v) the WIT 30 min + CS 2 h + HMP 2 h + SP600125 group (*n* = 12), in which the DCD modeled pigs were subjected to warm ischemia for 30 min and cold preserved for 2 h, after which 20 µM SP600125 was added to the preservation/perfusion solution, followed by 2 h HMP and liver transplantation. No immunosuppressants were used in the liver transplantation procedure. In the sham group, the pigs were numbered as 1, 2, 3, 4, 5, and 6, and in the WIT 30 min + CS 4 h DCD group, the pigs were numbered as 7, 8, 9, 10, 11, 12, 13, 14, 15, 16, 17, 18, etc. We randomly specified a point in a random number table to record pig numbers in horizontal order. The random number of each block was divided by 5, leaving a remainder of 0, 1, 2, 3, or 4. The pigs with remainder 0 were assigned to the sham group; the remainder 1 was assigned to the WIT 30 min + CS 4 h DCD group, the remainder 2 was assigned to the WIT 30 min + CS 4 h + SP600125 DCD group, the remainder 3 was assigned to the WIT 30 min + CS 2 h + HMP 2 h DCD group, and the remainder 4 was assigned to the WIT 30 min + CS 2 h + HMP 2 h + SP600125 group. If the remainders of two consecutive numbers were the same, the allocation of the latter number would be one group less. When a group was completed, the remaining pig was assigned to the last group.

The DCD model was established as previously described [9, 20]. All pigs were sent to the laboratory 1 week before the experiment for acclimation, thus minimizing confounds due to physiological stress reactions. The pigs were subjected to 12-h fast and 6-h water deprivation prior to operation. They were the anesthetized through the pump-controlled intravenous injection of propofol and cisatracurium, with the simultaneous inhalation of isoflurane, along with mechanical ventilation through an endotracheal tube. The right internal jugular vein and the right common carotid artery were exposed through a transverse incision on the neck, and a central venous catheter and arterial catheter were inserted, to be used for blood collection, drug administration, and monitoring. After this surgical procedure, the ventilator was removed and warm ischemia was initiated when mean arterial pressure (MAP) was < 60 mmHg. The presence of cardiac arrest or MAP < 25 mmHg and pulse pressure < 20 mmHg indicated cardiac death. The liver was collected from each subject when warm ischemia had lasted for the designated period.

### Collection and preparation of donor livers

Abdominal organs were collected using the rapid en bloc technique for liver and pancreas procurement [21]. Following entry to the abdomen, the liver quality was detected by visual inspection. Once liver function had been confirmed to be sufficient for liver transplantation, the abdominal aorta was isolated and superior mesenteric vein was ligated. Then, 30 min after onset of warm ischemia, LPS-1 solution (based on the formula for KPS-1, 1000 mL, 4 °C) was perfused through the abdominal aorta and superior mesenteric vein via gravity perfusion at a height of 0.8 m. The composition of KPS-1 (Organ Recovery Systems Inc., Itasca, IL, USA) and the additional agents added to produce LPS-1 are provided in Table 1. Ice chips were placed around the liver to decrease it temperature quickly. The gall bladder was cut open to remove the bile. The gastrointestinal tract and corresponding mesenterium were freed. The biliary tract was washed with the use of 50 mL LPS-1, which was poured via the lower segment of the bile duct. Subsequently, the liver, kidneys, pancreas, and spleen were harvested and stored in a sterile container containing LPS-1 at 4 °C. The suprahepatic inferior vena cava, infrahepatic vena cava, portal vein, hepatic artery, and common bile duct were adjusted to the appropriate lengths for machine perfusion attachment. Next, kidneys, pancreas, spleen, and excess muscle, along with adipose tissues were removed. For the HMP treatment, donor livers were placed in the portal vein and hepatic artery casing prior to beginning the perfusion.

**Table 1:** Composition of KPS-1 and agents added to produce LPS-1

Ingredients	Amount
KPS-1	1 L
6N HCl (as a pH preadjustment additive)	1.6 mL
α-Ketoglutaric acid	2 mg
*N*-Acetylcysteine	2 mg
l-Arginine	2 g
Nitroglycerin	50 mg
Alprostadil	2 vials (concentration, 500 μg/mL)

### HMP

The Lifeport Liver Transporter (Organ Recovery Systems Inc., Itasca, IL, USA) was introduced for HMP (Additional file 1: Fig. S1). The tubes were connected with reference to the operating procedure, and machine perfusion was initiated after organ storage on ice. The portal vein and hepatic artery were connected to the perfusion tube followed by HMP, with the perfusion rate adjusted according to the liver weight (0.667 mL/g liver/min). Any sudden increase in perfusion pressure caused by the folding of tubes triggered an alarm, and was promptly corrected.

### Recipient liver transplantation model

Classic orthotopic liver transplantation without venovenous bypass was performed on pigs in the experimental groups [22]. After Mercedes incision laparotomy, the liver was freed from its attachments. The hepatoduodenal ligament was freed and resected to isolate the common bile duct, hepatic artery, and portal vein. The common bile duct and hepatic artery were ligated and cut near the liver. Subsequently, the suprahepatic inferior vena cava and infrahepatic vena cava were isolated, followed by casing. Three phrenic veins were sutured and ligatured close to suprahepatic inferior vena cava. The infrahepatic vena cava and portal vein were experimentally pre-blocked three times under ordinary circumstances through colloid supply and norepinephrine application to increase blood pressure as required. When blood pressure and pulse rate were stable, the portal vein, suprahepatic inferior vena cava, and infrahepatic vena cava were blocked, followed by the removal of recipient livers, whereupon the anhepatic phase began. At this point, methylprednisolone (500 mg) was intravenously injected to alleviate inflammatory reaction in the recipient pigs, whereupon the colloid was quickly supplied and the amount of norepinephrine was adjusted according to present blood pressure. The restoration of blood flow to the liver following the anastomosis of suprahepatic inferior vena cava and portal vein indicated the end of the anhepatic phase. Subsequently, calcium gluconate (20 mL, 10%), sodium bicarbonate (100 mL), and methylprednisolone (500 mg) were injected. The transplanted liver was washed in saline water for rapid rewarming, and the infrahepatic vena cava, hepatic artery, and common bile duct were anastomosed, with end-to-end anastomosis for the common bile duct. Then, a silicon tube was placed in the bile duct to serve as a stent for fixation. Once substantial abdominal bleeding had stopped, the abdominal cavity was closed, and the pigs were allowed to recover.

### Tissue sampling and analysis

The animals were followed up to 5 days following transplant. Venous and arterial blood samples were collected from the catheters after opening of the abdomen of recipient livers in the sham and transplantation groups, at 1 h after blood restoration of hepatic artery, and at 1, 2, 3, 4, and 5 days after the operation for further serological examination. Blood was immediately centrifuged, and the plasma was frozen and stored at − 20 °C until analysis.

Appropriate portions of liver tissues were collected through relaparotomy from recipient livers before warm ischemia, 30 min after warm ischemia, 2 h after cold storage, 4 h after cold storage, 2 h after HMP, and 24 h after transplantation. The biopsy samples were either stored at − 80 °C or preserved in 10% formaldehyde for further analysis. At the end of the study, the animals were tranquilized and then euthanized by injection of an overdose of pentobarbital sodium (> 150 mg/kg) after establishment of an intravenous passage.

### Biochemical assay of blood sample

The dry chemistry method was applied to determine glutamic pyruvic transaminase (ALT), glutamic oxaloacetic transaminase (AST), and total bilirubin (TBIL) using the Vitros 5600 Automatic Biochemical Immunoanalyzer (Johnson & Johnson, New Brunswick, NJ, USA). The international normalized ratio (INR) was measured by immunomagnetic beads using an automatic coagulometer (Stago, France). The lactate was analyzed by GEM 3500 blood electrolyte analyzer.

### Histological evaluation

Liver tissues were immediately fixed with 10% neutral formaldehyde. Subsequently, the tissues were dehydrated, embedded in paraffin, sliced, and stained with HE. Apoptosis of the liver tissues was detected using the terminal deoxynucleotidyl transferase mediated 2′-deoxyuridine 5′-triphosphate nick-end labeling (TUNEL) assay according to the instructions of the assay kit (Beyotime, Ningbo, Zhejiang, China). Histopathologists who were blinded to the experimental condition assessed the extent of tissue damage of the samples. Liver injury was scored regarding sinusoidal congestion, vacuolization of hepatocyte cytoplasm, and parenchyma, as previously reported [23–25].

### Western blot analysis

Proteins obtained from liver tissues were analyzed by Western blot analysis. Blots underwent incubation with antibodies against JNK (Anti-JNK1/2/3 antibody bs-2592R; Bioss, Beijing, China), phosphorylated-JNK (P-JNK) (Anti-phospho-JNK1/2/3 antibody bs-1640R; Bioss), B-cell lymphoma-2 (Bcl-2) (Anti-Bcl-2 antibody ab117115; Abcam Inc., Cambridge, MA, UK), Bcl-2 associated protein X (Bax) (Anti-Bax antibody bs-0127R; Bioss), Cleaved Caspase-3 (Anti-Cleaved Caspase-3 ab49822; Abcam), Cytochrome C (Anti-Cytochrome C antibody ab90529; Abcam), and glyceraldehyde-3-phosphate dehydrogenase (GAPDH) (Anti-GAPDH antibody ab8245; Abcam). The blots were scanned with ChemiDoc™ XRS (Bio-Rad, USA). Image J 7.0 software was used for the analysis of the gray area density.

### Statistical analysis

Data were analyzed with the SPSS 16.0 software and presented as the mean ± standard deviation values. The two-way analysis of variance (ANOVA) was used for the comparison of variables among 5 groups. Differences between 2 groups were analyzed with the 2-tailed Student’s *t *test, while the Chi-square test was applied for categorical variables. *p* < 0.05 difference was considered statistically significant.

## Results

### Comparison of the effects of cold storage and HMP on surgical parameters

There were no significant group differences in the weights of donor and recipient pigs, cold storage period, surgical time of recipient pigs, anhepatic phase, intraoperative blood loss, period between anhepatic phase and inferior vena cava opening, and period between inferior vena cava opening and hepatic artery, as shown in Table 2 (*p* > 0.05).

**Table 2:** The comparative protective effects of cold storage and HMP on surgical parameters

	CS (*n* = 6)	CS + SP600125 (*n* = 6)	HMP (*n* = 6)	HMP + SP600125 (*n* = 6)
Weight of donor pigs (kg)	35.20 ± 2.89	36.50 ± 2.78	34.40 ± 2.87	33.40 ± 2.34
Weight of recipient pigs (kg)	37.30 ± 3.09	37.20 ± 2.85	36.70 ± 3.02	35.80 ± 2.21
Surgical time of recipient pigs (min)	211.30 ± 37.25	203.00 ± 32.44	215.80 ± 40.43	212.78 ± 36.21
Anhepatic phase (min)	28.30 ± 2.76	28.10 ± 2.99	27.70 ± 2.34	29.10 ± 1.97
Intraoperative blood loss (mL)	583.20 ± 148.33	533.60 ± 164.33	601.30 ± 180.22	502.37 ± 163.22
Period between anhepatic phase and inferior vena cava opening (min)	45.30 ± 4.35	46.20 ± 3.01	43.80 ± 4.88	44.20 ± 4.21
Period between inferior vena cava opening and hepatic artery opening (min)	30.40 ± 5.23	29.30 ± 4.76	28.30 ± 3.43	27.80 ± 4.03

*CS* cold storage, *HMP* hypothermic machine perfusion

### Comparison of the effects of cold storage and HMP on perfusion parameters

The perfusion parameters in the Lifeport system were stable in the HMP groups during the perfusion period (Additional file 2: Fig. S2).

### Comparison of the effects of cold storage and HMP on serological examination

**Fig. 1:**
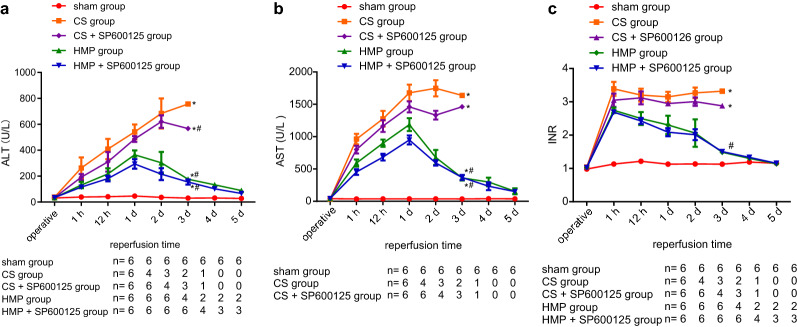
The comparative protective effects of cold storage and HMP regarding serological examination results. **a** ALT in each group. **b** AST in each group. **c** INR in each group. * Indicates *p* < 0.05 compared with sham group at third day; # indicates *p* < 0.05 compared with CS group at the second day

The ALT concentration of each group in the perioperative period is depicted in Fig. 1a, showing that that ALT levels began to rise in the CS and CS + SP600125 groups following transplantation, and that all these recipients died within 3 days. In the HMP and HMP + SP600125 groups, ALT reached its peak on the first day and gradually decreased thereafter. On the 5th day, ALT of all the surviving recipients had decreased to the normal range. AST levels of each group recorded in the perioperative period are shown in Fig. 1b. The time course of AST changes matched the findings for ALT. Similar changes were seen for AST, showing a rise in AST in the CS and CS + SP600125 groups after transplantation, with all these recipients dying within 3 days. In the HMP and HMP + SP600125 groups, AST peaked on the first day and gradually decreased afterwards. On the 5th day, ALT of surviving recipients had decreased to normal level. Figure 1c illustrates the coagulation index (INR) of each group in the perioperative period. After transplantation, INR remained elevated, without any decrease in the CS and CS + SP600125 groups, while a gradual reduction was detected in the HMP and HMP + SP600125 groups. The INR of the surviving recipients had declined to the normal range on the 5th day.

**Fig. 2:**
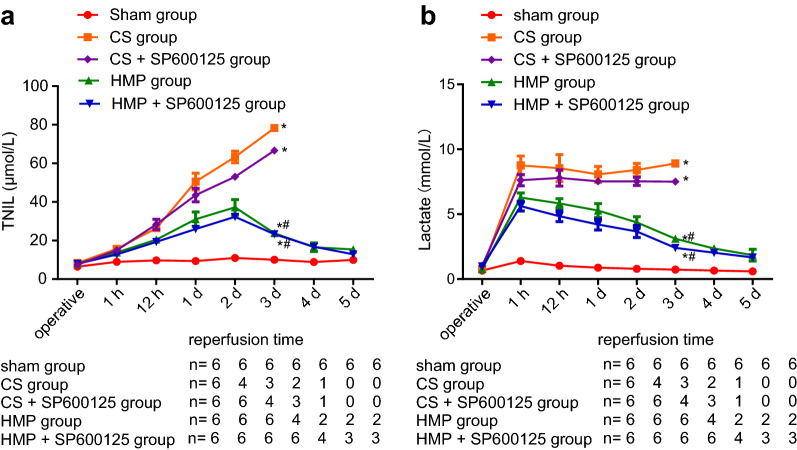
The comparative protective effects of cold storage and HMP on plasma TBIL and lactate in each group. **a** TBIL in each group. **b** Lactate concentration in each group. * Indicates *p* < 0.05 compared with sham group at day 3; # indicates *p* < 0.05 compared with CS group at day 2

As shown in Fig. 2a, TBIL continued to increase in the CS and CS + SP600125 groups during the perioperative period, and none of these recipients survived for more than 3 days after transplantation. TBIL peaked on the 2nd day, whereupon it began to decline in the HMP and HMP + SP600125 groups, while TBIL of survivors had declined to the normal range on day 5. Figure 2b illustrates the findings of plasma lactate levels during the perioperative period. Following transplantation, lactate remained at a high level in the CS and CS + SP600125 groups and did not decline. In the HMP and HMP + SP600125 groups, lactate began to decline, returning to normal on the 5th day in surviving recipients.

### Comparison of the effects of cold storage and HMP on histopathological changes

**Fig. 3:**
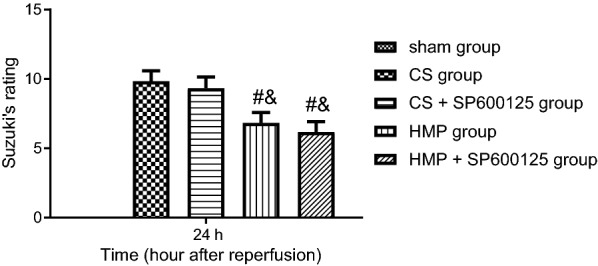
The comparative protective effects of cold storage and HMP on histopathological changes of liver tissues. Suzuki’s rating at 24 h post transplantation. ^#^*p* < 0.05 vs. the CS group, ^&^*p* < 0.05 vs. the CS + SP600125 group

The results of HE staining of samples from donor livers with warm ischemia, cold storage, and HMP treatments are shown in Additional file 3: Fig. S3A. In the sham group, the hepatic lobule was well formed, hepatocytes were clearly structured, and the portal area was normal, without the presence of evident edema or congestion in the hepatic sinusoid and blood vessels. Following 30-min warm ischemia, the hepatic lobule was still intact, while edema to different degrees had developed in liver cells with relatively more vacuolar degeneration, obvious congestion, and scattered necrosis. After warm ischemia for 30 min and cold storage for 2 h, the damage was not significantly aggravated, but there was much more vacuolar degeneration, with or without addition of SP600125 in the storage medium. However, when cold storage was elongated for an additional 2 h, the damage was aggravated, vacuolar degeneration was promoted, and there was obvious necrosis in some areas with or without addition of SP600125. Furthermore, the application of HMP for 2 h resulted in more severe edema, but alleviated vacuolar degeneration, and led to almost no necrosis. As depicted in Additional file 3: Fig. S3B, to further compare the role of cold storage and HMP in liver injury, we applied HE staining to donor livers at 24 h after transplantation in different treatment groups. Severe congestion was observed in the CS and CS + SP600125 groups, in which edema and vacuolar degeneration were aggravated and there was partial loss of the hepatic lobule. Compared with cold storage treatment, the HMP and HMP + SP600125 groups presented with mild congestion, edema and vacuolar degeneration, with intact hepatic lobules without obvious necrosis, thus indicating a significant alleviation of liver damage. The difference of damage was further quantified with the use of Suzuki’s rating (Fig. 3), which showed a significant difference between cold storage and HMP treatments (*p* < 0.05), but no significant difference between the HMP and HMP + SP600125 groups (*p* > 0.05).

### Comparison of the effects of cold storage and HMP on expression of JNK and P-JNK

**Fig. 4:**
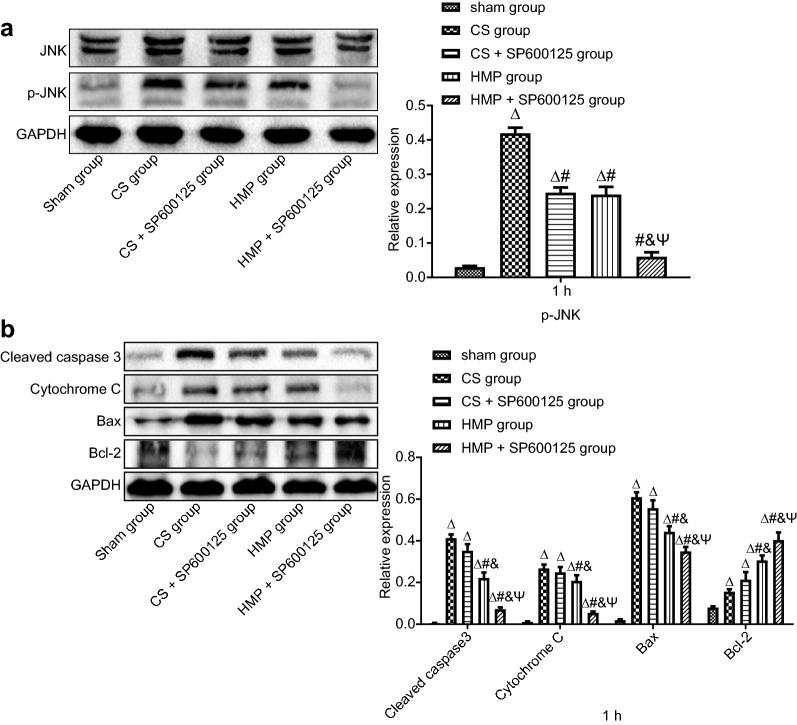
The comparative protective effects of cold storage and HMP on expression of JNK and P-JNK. **a**, **b** Relative protein expression of JNK and P-JNK normalized to GAPDH in each group after the blood flow was restored for 1 h determined by Western blot analysis. **b** Relative expression of apoptosis-related proteins (Cleaved Caspase-3, Cytochrome C, Bax and Bcl-2) normalized to GAPDH in each group after the blood flow was restored for 1 h determined by Western blot analysis. ^∆^*p* < 0.05 vs. the sham group, ^#^*p* < 0.05 vs. the CS group, ^&^*p* < 0.05 vs. the CS + SP600125 group, ^Ψ^*p* < 0.05 vs. the HMP group

Western blot analysis shows the protein expression of JNK in each group after the blood flow was restored for 1 h (Fig. 4a). These results show that P-JNK expression was significantly elevated in the CS group compared to the sham group (*p* < 0.05), while P-JNK expression was non-significantly lower in the CS + SP600125 and HMP groups than in the CS group (*p* > 0.05). However, P-JNK was significantly diminished in the HMP + SP600125 group when compared with the CS, CS + SP600125, and HMP groups (*p* < 0.05). The relative expression of apoptosis-related proteins in each group after the blood flow was restored for 1 h is shown in Fig. 4b. The results reveal a significant increase in the expression of pro-apoptotic proteins (Cleaved Caspase-3, Cytochrome C, and Bax) in the CS and CS + SP600125 groups in comparison to the HMP and HMP + SP600125 groups (*p* < 0.05), but no significant differences between the CS and CS + SP600125 groups, with the CS + SP600125 group revealing lower expression (*p* > 0.05). The difference in apoptotic markers between the HMP and HMP + SP600125 groups was significant, with the HMP + SP600125 group presenting with decreased expression of pro-apoptotic proteins. Opposite findings were observed for the expression of an anti-apoptotic protein (Bcl-2).

### Comparison of the effects of cold storage and HMP on apoptosis

**Fig. 5:**
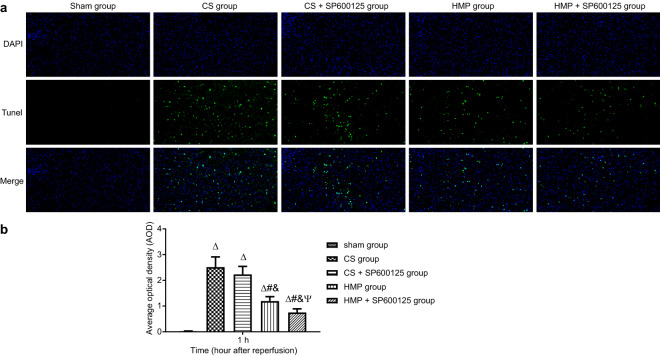
The comparative protective effects of cold storage and HMP on apoptosis in each group determined by TUNEL staining. **a** TUNEL assay performed on donor liver tissues in each group. **b** Average optical density in each group. ^∆^*p* < 0.05 vs. the sham group, ^#^*p* < 0.05 vs. the CS group, ^&^*p* < 0.05 vs. the CS + SP600125 group, ^Ψ^*p* < 0.05 vs. the HMP group

The TUNEL assay was performed at 24 h after transplantation in each group (Fig. 5a, b). There was no significant difference detected between the CS and CS + SP600125 group (*p* > 0.05), while a significant difference was present between the HMP and HMP + SP600125 groups, as well as between CS and HMP treatment groups (*p* < 0.05). The TUNEL results were proportional with the degree of openness of the mitochondrial permeability transition pore, indicating that HMP could result in the suppression of apoptosis after transplantation compared to the use of cold storage, and that further addition of SP600125 in the perfusion solution exerted greater inhibitory effects on apoptosis.

### Comparison of the effects of cold storage and HMP on survival analysis

**Fig. 6:**
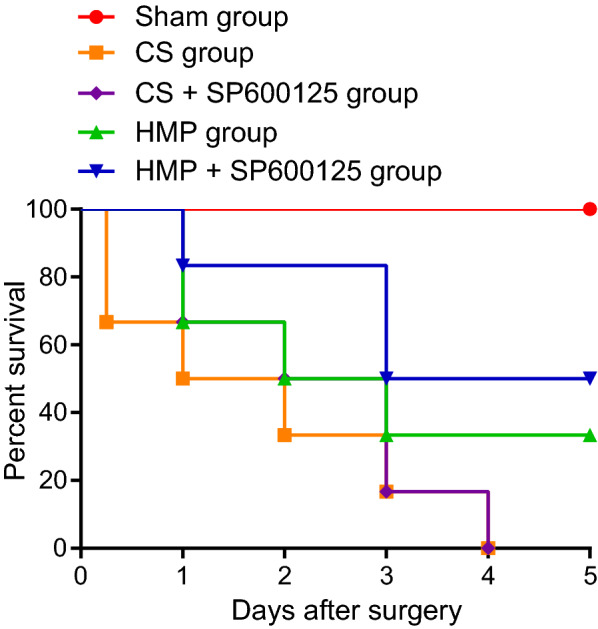
The comparative protective effects of cold storage and HMP on 5 day survival rates in each group after the operation

The survival rates are plotted in Fig. 6. Results revealed that the 5-day survival rate of the sham group was 100%, versus 50% for the HMP + SP600125 group, 33% for the HMP group, and 0% for the CS and CS + SP600125 groups. The overwhelming majority of recipients died within 3 days after transplantation. The analysis from log-rank testing indicated a significant difference between the CS and HMP + SP600125 group (*p* = 0.038), while the difference in survival among other groups after transplantation was insignificant (*p* > 0.05).

## Discussion

In the present study, we evaluated the protective effects of HMP in combination with SP600125 (a specific JNK signaling pathway inhibitor) on the quality of marginal donor livers from DCD following liver transplantation in pigs. The findings revealed that the application of HMP following 2-h cold storage could alleviate IRI in pigs after liver transplantation, thus improving the survival. Moreover, the addition of SP600125 in the HMP perfusion solution further alleviated IRI and inhibited apoptosis, thereby playing a potential protective role for marginally viable donor livers.

In the current investigation, while the period of warm ischemia was maintained at the same level, the storage modes differed between groups of recipient pigs. Results confirmed a protective effect of HMP on marginally viable donor livers in comparison to the use of cold storage alone. Accumulating animal experiments have provided a body of evidence proving the advantages presented by HMP for the improved viability of donor livers used in liver transplantation [8, 26–29]. The continuous circumfusion maintains the microcirculatory homeostasis of the removed liver, maintaining sufficient of supplies of oxygen and nutrients to fulfil the immediate metabolic requirements of the organ. The function and quality of organs can be evaluated by detecting metabolites in the outflowing perfusion solution, which reveals the clearance metabolic wastes and toxic substances, showing that supplementation with cytoprotective substances and drugs can improve the quality of donor livers. Taken together, our results show that HMP can increase the survival rate of grafts, thus improving the recipient’s prognosis. Although the superiority of HMP in the late phase of ischemia has been confirmed, its optimal application time is still being debated [30, 31]. In our study, HMP was applied for 2 h in the late phase of ischemia, which is consistent with clinical practice, as reported in a previous study [32].

Different organs have varying capacities for tolerating warm ischemia. It is generally agreed that human livers can tolerate warm ischemia for at most 30 min, and that longer periods bring a much higher risk of primary failure of donor livers following liver transplantation [33]. In this study, we compared between different storage modes for marginal donor livers in a pig DCD model, in which we used the human/clinical threshold of 30 min of warm ischemia for donor liver transplantation. Histological results revealed varying degrees of edema in liver cells, accompanied by more vacuolar degeneration, obvious congestion, and scattered necrosis following 30-min warm ischemia, all of which findings are in line with a previous report [34]. After 2-h cold storage, the degree of observed congestion was improved by cold perfusion, while other cellular injuries were not significantly aggravated. However, after 4-h cold storage, the injuries were aggravated in parallel with exacerbated vacuolar degeneration and obvious necrosis in some areas, irrespective of the presence or absence of SP600125. Furthermore, the application of HMP for 2 h following 2-h cold storage resulted in a more severe edema, but with alleviated vacuolar degeneration and almost no necrosis, with or without SP600125 treatment. The aforementioned results indicate that a short-term HMP imparts satisfactory storage effects on marginal donor livers, which is supported by results from basic and clinical research [29, 35].

After transplantation, we saw significant group differences in serological markers, pathological results, and survival rate after the use of HMP in comparison to cold storage at 24 h after the restoration of perfusion. Previous studies have demonstrated that the supplementation of specific drugs in the perfusion solution during HMP can promote the recovery of stressed donor livers or indeed alleviate IRI [36, 37]. The significance of JNK for the MAPK signaling pathway for liver IRI has been highlighted in previous studies [38]. It is known that SP600125 alleviates liver IRI through the inhibition of the JNK signaling pathway, which leads to suppressed apoptosis in rat and mouse models [14, 15, 17]. The direct application of the specific NMK inhibitor SP600125 to donors and recipients in simulations of clinical situations is inappropriate, due to the systemic toxicity. In this study, SP600125 was instead added to the perfusion solution for HMP, which showed a substantial effect regarding the extent of JNK phosphorylation and liver cell apoptosis in comparison with the use of HMP alone. Moreover, the extent of JNK phosphorylation also significantly differed between the CS and CS + SP600125 groups at 1 h after the blood flow was restored, yet apoptosis, pathological results, serological examination and survival rate did not differ significantly, suggesting that IRI following liver transplantation is a much more complex condition than is explicable by the JNK signaling pathway alone.

Present results nonetheless indicate that the use of HMP in combination with SP600125 can significantly improve the prognosis of DCD for liver transplantation. However, we note some limitations of the present study. First, we have not established an optimal concentration of SP600125 in the perfusion solution, in part due to the present lack of reports giving guidelines or instructions on this issue. Based on previous animal studies, we applied a concentration of 20 µM in the preservation/perfusion solution [15], but this concentration may not be optimal with respect the benefits versus toxic side effect profile. Second, due to limitations in our experimental conditions, we only followed our transplant pigs for 5 days following the operation, which was not long enough to assess and compare the differences in prognosis between the HMP and HMP + SP600125 groups, including long-term survival rate and long-term complications like ischemic bile duct diseases.

## Conclusion

HMP during the last phase of cold ischemia can improve the quality of donor livers from DCD and improve the prognosis following liver transplantation. Moreover, the addition of SP600125 in the HMP perfusion solution can result in the inactivation of the JNK signaling pathway and suppression of apoptosis, thus alleviating IRI of donor livers and contributing to better quality of donor livers. Since the toxic side effects of SP600125 limit its systemic use for human donors or recipients, we undertook the present pig study to confirm the protective effects of HMP combined with SP600125 in the donor liver perfusion solution. Therefore, our study provides a theoretical basis for improved treatment, while not yet being directly translatable into clinical practice. Previous research has shown that SP600125 has developmental toxicity, which may be a further consideration limiting its translational use [39]. Results of this and previous animal studies show that SP600125 can be perfused before liver transplantation [40–42]. Although experimental animals such as mice and pigs are of great reference value for human transplantation, better judgement of the merits of our present approach call for further investigations of toxicity and optimal medication doses.

## Supplementary Information

**Additional file 1: Figure S1.** The design of HMP.

**Additional file 2: Figure S2.** The comparative protective effects of cold storage and HMP on perfusion parameters of HMP.

**Additional file 3: Figure S3.** The comparative protective effects of cold storage and HMP on histopathological changes of liver tissues. A, HE staining of donor liver samples during warm ischemia, cold storage and HMP in various groups (×100). B, HE staining of donor livers at 24 h post transplantation in various groups (×100, ×200).

## Data Availability

Data generated and analyzed as part of this study are included in the manuscript or are available upon request from the corresponding author.

## Abbreviations

HMP: Hypothermic machine perfusion
JNK: C-Jun N-terminal kinase
IRI: Ischemia–reperfusion injury
CS4h: Cold storage for 4 h
DCD: Donation after cardiac death
MAPK: Mitogen-activated protein kinase
MAP: Mean arterial pressure
ALT: Glutamic pyruvic transaminase
AST: Glutamic oxaloacetic transaminase
TBIL: Total bilirubin
INR: International normalized ratio
HE: Hematoxylin–eosin
ANOVA: Analysis of variance
DBD: Donation after brain death
